# Isolated Sphenoid Sinusitis: Anatomical Features for Choosing a Method of Treatment, a Case-Control Study

**DOI:** 10.3390/diagnostics12051284

**Published:** 2022-05-21

**Authors:** Sergei Karpishchenko, Olga Vereshchagina, Olga Stancheva, Tatiana Nagornykh, Alexander Krasichkov, Irina Serdiukova, Aleksandr Sinitca, Dmitry Kaplun

**Affiliations:** 1 ENT Department with Clinic, Pavlov First Saint Petersburg State Medical University, 195176 St. Petersburg, Russia; karpischenkos@mail.ru (S.K.); wereschagina@yandex.ru (O.V.); tanya-nago@mail.ru (T.N.); 2 Radio Engineering Systems Department, Saint Petersburg Electrotechnical University “LETI”, 197022 St. Petersburg, Russia; askrasichkov@etu.ru (A.K.); i.a.serdiukova@gmail.com (I.S.); 3 Research Centre for Digital Telecommunication Technologies, Saint Petersburg Electrotechnical University ”LETI”, 197022 St. Petersburg, Russia; amsinitca@etu.ru; 4 Faculty of Information Technologies, Kazakh-British Technical University, Almaty 050000, Kazakhstan; dikaplun@etu.ru; 5 Department of Automation and Control Processes, Saint Petersburg Electrotechnical University “LETI”, 197022 St. Petersburg, Russia

**Keywords:** sphenoid sinus, endoscopy, computed tomography, nasal cavity, paranasal sinuses

## Abstract

Isolated sphenoid sinusitis (ISS) is a group of pathologies characterized by inflammation in one or both sphenoid sinuses. The gold standard for analyzing and diagnosing ISS is computer tomography. Many researchers have discussed the treatment of patients with ISS variants such as fully opacified sinus, mostly with surgery. A retrospective analysis of clinical data of 59 patients (21 male (35%), 38 female (65%)) with ISS, who were treated in the Otorhinolaryngological Department of Pavlov First Saint Petersburg State Medical University between January 2017 and January 2020, was conducted. All patients were in the first stage of the same medical therapy. In cases where there was no recovery, these patients were referred to surgery. For the control group, we analyzed patients without any disorders according to CT-scan examination. After analyzing the obtained clinical and radiological data, we found indicators that were common in patients who did not recover after medical therapy. According to the reverse regression method statistical model, in male patients with a diffuse headache and nasal discharge it was shown that medical therapy was highly effective (more than 78%). The presence of nasal septum deviation and adenoids in male and female patients leads to the highest risk of surgical treatment (83% probability of the logistic model). The detailed analysis of CT-scans and the complaints of patients with ISS can be the key to determining the preferred therapy choice. Not all cases need to have an endoscopic opening of the sphenoid sinus, according to our research.

## 1. Introduction

Sphenoid sinus disease is a pathology which is characterized by inflammation of the sphenoid sinus mucosa. As a rule, such changes in the sphenoid sinus are usually combined with another rhinological pathology. From all paranasal sinus pathologies, isolated sinusitis of the sphenoid occurs in 1–3% of all inflammatory cases [1]. The anatomical and topographical locations of the sphenoid sinus, as well as non-specific symptoms, result in late diagnosis of this disease. Among all complaints, the most common is headache in the retro-orbital and occipital areas (72%) [2]. The second most common complaint involves located visual disturbances such as diplopia, a progressive unilateral vision loss, and depressing of the visual fields on the side of the lesion. Ophthalmological symptoms accounted for 21% of patients with isolated sphenoid sinusitis [3]. Rhinological manifestations of the disease include post-nasal drip and epistaxis. Thus, patients are under the supervision of doctors of related specialties for a long time, such as ophthalmologists, neurologists and other specialists [4].

Diagnosis of isolated sphenoid sinusitis is based on anamnesis, endoscopic examination of the sphenoethmoidal area, and also on computer and magnetic resonance imaging [5]. The last study is usually prescribed by neurologists as an additional diagnostic method for unknown cephalgia [6]. The gold standard in the diagnosis of ISS is computed tomography of the sinuses [7]. With the use of this method, it is possible to assess the bony borders of the sinus and adjacent intranasal structures [8]. The existing nasal septum deviation, or hypertrophy of the middle or superior turbinate, can predispose a patient to hypoventilation of the sinus, and a violation of its drainage function. In such a situation, the delivery of drugs directly to the sinus will be difficult and ineffective [5].

Primary assessment revealed that total opacification of the sphenoid according CT-scans with clinical manifestations should be treated with the use of saline irrigation, antibiotics, topical corticosteroids and surgical interventions, if there are indications [1]. There is a well-known technique of irrigation using the Dolphin system, during which the patient is in the nose-to-ceiling and then the nose-to floor head position [9]. Such a method is proven to be useful for sphenoid sinus irrigation. In non-complicated cases, after 7–14 days of treatment the control CT-scan is allowed to be performed [10]. Absence of recovery indicates that surgical intervention is needed.

There are several approaches for treating the sphenoid sinus opening. The most common of them are the following: endoscopic endonasal, endoscopic transseptal and endoscopic transethmoidal approaches [8,11]. In the case of an isolated pathology of the sphenoid sinus, the first two methods are considered the most optimal, since they do not require destruction of other intranasal structures. If the patient has a combination of sphenoid sinus hyperpneumatization and inflammation, especially in lateral parts of the sinus, the risk of damaging the vital structures during surgery increases [12]. In order to prevent the development of complications during surgery, electromagnetic navigation control as an additional method can be used. Such a method allows high-precision identification of “dangerous” areas inside the sinus [4].

We analyzed patients with ISS, their medical histories and CT-scans in order to develop a clinically and anatomically grounded approach to the treatment of choice. There are no data in the literature that consider anatomical structures, which lead to hypoventilation as a reason for choosing a method of treatment.

## 2. Materials and Methods

We investigated patients diagnosed with ISS in the ENT Department at Pavlov First Saint Petersburg State Medical University, Russia. The study was approved by the Ethics Committee of the Pavlov First Saint Petersburg State Medical University (17 November 2017, protocol No. 11). Written informed consent was obtained from all participants.

A prospective study was performed that involved patients with a diagnosis of isolated sphenoid sinusitis. In total, 59 people, aged 4–68 years, took part in the study. The study was conducted in the pediatric department and in the adult department of otorhinolaryngology. In order to provide case-control research in the field of isolated bacterial sphenoid sinusitis, the exclusion criteria from this research were neoplasm, intracranial involvement of pathological process, pregnancy, and a history of previous ISS [13].

In all cases, patients were admitted to the hospital with subtotal opacification of one or both sphenoid sinuses, and normal pneumatization in others. All patients were admitted to our department on an emergency basis, and the only available rapid diagnostic method that we had was CT. In the event of a diagnosis, we did not repeat the MRI. Patients who had an MRI were referred to us for emergency hospitalization from neurologists and ophthalmologists. At the initial stage, all patients had the same medical therapy (systemic decongestant, systemic antibacterial therapy, topical glucocorticosteroids, oral corticosteroids and nose irrigation) [14,15].

**Figure 1:**
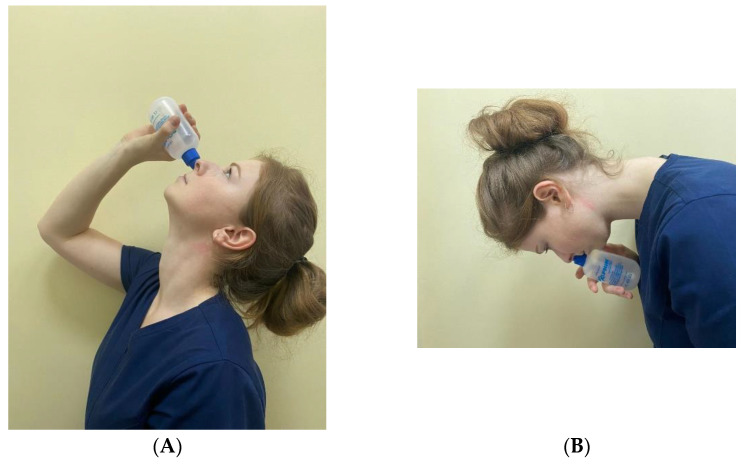
Example of the nose-to-ceiling (**A**) and nose-to-floor (**B**) head positions during the irrigation.

Irrigation included the special technique that used the Dolphin system, which allows the delivery of the saline solution to the sinus (Figure 1).

Then, after 14 days, the dynamics of treatment were assessed with the use of computed tomography of the paranasal sinuses [16,17].

**Figure 2:**
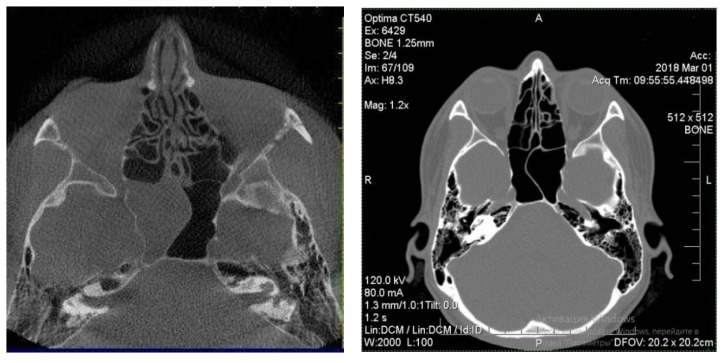
CT-scan of a patient, who showed good outcomes after conservative treatment and was added to group 1.

Patients with good outcomes after the treatment, which included residual edematous changes of the sinus mucosa on the CT scan, were designated as group 1 of the study (Figure 2) [18]. This group included 22 patients aged from 6 to 68 years old.

Patients whose CT scan results did not differ from the initial data nor showed negative dynamics such as involvement in the inflammatory process of posterior ethmoidal cells on the affected site, were referred for surgical treatment (endoscopic sphenoidotomy) [19]. These patients constituted group 2 of the study, and named the ineffective conservative treatment group. In total, the second group included 37 patients aged from 4 to 68 years old.

The control group consisted of patients who had no pathological changes according to CT scans. The last group consisted of 33 patients aged 8–70 years. These were patients who underwent CT scans because of ophthalmological indications. Most of them had dacryostenosis, and contrasting of the lacrimal pathways. It was these patients who had no nasal complaints and underwent CT.

All subjects of groups 1 and 2 underwent a comprehensive examination, which included:

- Otorhinolaryngological examination (*n* = 59).
- Rigid endoscopy of the nasal cavity and nasopharynx (*n* = 59).
- Computed tomography of the paranasal sinuses (*n* = 92).
- Magnetic resonance imaging of the paranasal sinuses (*n* = 6)
- Bacteriological examination from the sphenoid sinus directly (*n* = 37).
- Bacteriological examination of the nasal cavity mucus (*n* = 37).
- Measurement of the sphenoid sinus volume (before surgery/ during the surgery (*n* = 12)) [20].
- Measurement of the new formed sphenoid sinus osteum square (*n* = 37) [21].

To test whether the potential efficacy of therapeutic treatment can be predicted based on the patient’s clinical status determined by their anamnesis and objective measurements, a logistic regression model was utilized following a stepwise procedure outlined below.

Four models were constructed and analyzed: a step-by-step method of eliminating predictors, and a step-by-step method for including predictors, each considered with and without constant inclusion. As a result, the model built by the method of successive inclusion showed the greatest significance. This version of the model was chosen for construction by the forced inclusion method. The quality of the constructed model was determined by the Nagelkerke coefficients [22,23,24,25,26].

## 3. Results

**Figure 3:**
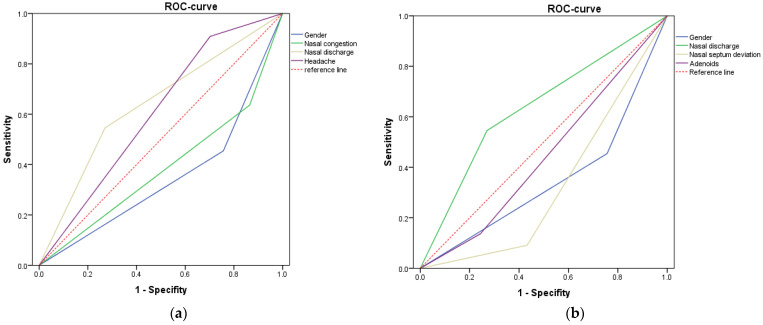
ROC curve for prediction of: (**a**) conservative treatment for complaints; (**b**) surgical therapy for complaints with pathology.

The effect of each of the potential predictors on the prediction of conservative treatment for complaints is shown in Figure 3a using an ROC curve. The ROC curve shows the effect of the variable on the prognosis of conservative treatment, the variables which are above the reference line confirming the hypothesis, and which variables below it that have the opposite effect [27,28,29,30]. The larger the area under the graph of the variable, the more influence it has on the model. Screening values of each marker can be found in Table 1 (group 1). For the resulting logistic regression model, a pair of expressions describes the probability of predicted type of therapy p:

p = 1/(1 + exp(−z))(1)

z1 = 2.571 − 2.185 × x1 − 2.166 × x2 + 2.274 × x3 − 2.455 × x4 + e(2)

where z1—is the regression Equation (2) based on the observed sample; x1—variable gender; x2—variable nasal discharge; x3—variable nasal congestion; x4—variable headache; and e—random errors in the construction of the model.

By analogy, the influence of each of the potential predictors on the prediction of surgical therapy for complaints with pathologies was assessed using the ROC curve (Figure 3b). Screening values of each marker can be found in Table 1 (group 2). The regression equation for this model is:

z2 = 1.274 − 2.852 × x1 + 2.233 × x2 + 2.769 × x3 − 1.809 × x4 + e(3)

where z2—is the regression Equation (3) based on the observed sample; x1—variable gender; x2—variable adenoids; x3—variable nasal septum deviation; x4—variable nasal discharge; and e—random errors in the construction of the model.

Thus, it was found that in male patients with a diffuse headache and with nasal discharge, the effectiveness of conservative therapy is high (about 78%), and the presence of the symptom of “nasal congestion” is highly likely to result in ineffective conservative treatment. In another model of logistic regression, taking into account the concomitant pathology of the nasal cavity, it was found that the presence of nasal septum deviation and adenoids in male and female patients leads to the need for surgical treatment of patients (83% probability of the logistic model).

**Figure 4:**
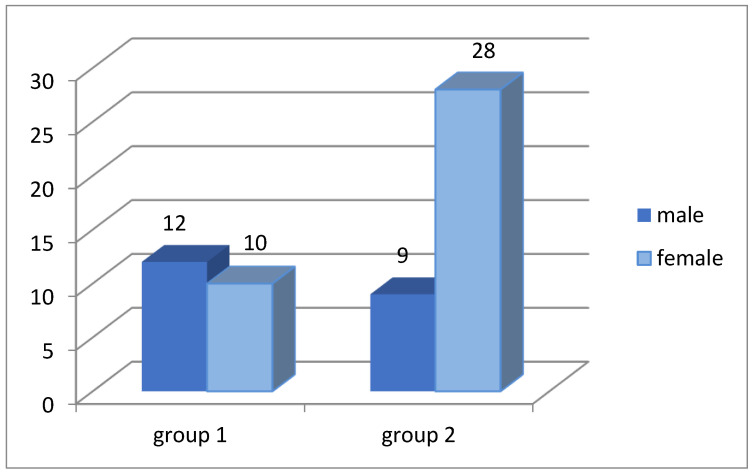
Diagram of gender distribution of patients with ISSf: group 1 with effective medical therapy, group 2 with unsuccessful medical therapy.

Age and gender distributions of patients characterized by a significant predominance of women in group 2 (ineffective conservative treatment) in comparison with group 1 (effective conservative treatment) are shown in Figure 4.

The median age of the patients of the two groups corresponds to group 1 Me = 14.5 years; group 2 Me = 28 years.

Among the two groups of patients, complaints of headache, postnasal drip and nasal congestion prevailed in patients with ineffective conservative treatment (Table 2).

During the analysis of the anatomical features of the intranasal structures in patients with isolated sphenoid sinusitis, it was found that in group 2, concomitant changes most often prevailed; this can lead to significant hypoventilation of the sphenoid sinus (Table 3). Deviated septum and adenoids were also common in the pediatric patient group, but surgical treatment was not always performed in these cases, since antibacterial therapy was highly effective. In deciding on the approach to treat sphenoid sinus, the presence of a nasal septal deviation on the side of the sphenoid sinus lesion should be considered. Thus, when combining the above factors, treatment preference is for the transseptal approach with simultaneous correction of the nasal septum deviation.

After sampling mucus from the sphenoid sinus, a comparative analysis of the bacterial composition of the nasal cavity and of the affected sphenoid sinus was performed (Table 4). Based on the data obtained, it follows that bacteriological analysis from the nasal cavity cannot serve as an indicator of the appointment of antibacterial drugs, taking into account sensitivity. This method showed us that the bacterial composition in the nasal cavity is different from the same phenomenon found in the sphenoid sinus.

In the second group of the study, patients with ineffective medical treatment that required surgical intervention, there were 12 patients with isolated fungal sinus disease. Since fungal infection is characterized by the highest recurrence rate, a method was developed to evaluate the effectiveness of surgical treatment of isolated fungal sphenoid sinusitis. The analysis consisted of comparing the volume of the affected sphenoid sinus according to computed tomography with the volume obtained by intraoperative measurement of the sinus volume. Intraoperative volume measurement includes the use of methylene blue solution, which was injected through an insulin syringe inside the sphenoid sinus until full. In the case of matching volumes, surgical removal of fungal masses from the sphenoid sinus was considered as complete. If the difference in volumes between the CT scans and the intraoperative liquid measurements is larger than 0.4 cm^3^, then not all fungal masses were removed; the surgeon should make a revision of the sphenoid sinus [20]. However, if the difference is less than 0.4 cm^3^, this indicates an edema of the sinus mucosa without pathological masses in the sinus. Furthermore, measurements of the volume of the sphenoid sinus made it possible to determine that isolated fungal balls are more common in patients with hyperpneumatization of the sphenoid sinus (the median of the volume was 6.8 cm^3^).

With the use of computed tomography, we compared the volumes of the involved sphenoid sinus between the two groups, as well as with the control group. The data showed no significant difference among all research groups, which means that the volume does not depend on the size of sinus with the development of the pathology in them.

## 4. Discussion

In recent times, we know of good medical treatment combinations for patients with ISS. This scheme includes systemic antibacterial therapy, topical corticosteroids and nasal irrigation. Craig et al. suggested a nose-to-ceiling head position for irrigation of the sphenoid sinus with saline solution. Such methods showed a good delivery of the topical therapy, according to the validated computational fluid dynamics (CFD) model [9]. It is unclear how in some cases that this combination is insufficient for recovery. Multiple studies have borne out the positive effects of endoscopic sphenoidotomy, which is thought to function by relieving ostial obstruction and thereby improve sinus ventilation [31]. Distribution of saline irrigations and other topical therapies have been limited in the unoperated state, and are likely even worse in the setting of inflammation and mucosal edema [32].

There is another rare group of patients with isolated mycotic sphenoid sinusitis who typically have a high percentage of recurrence, even when surgical interventions have been successful [33]. For this group of patients, it is necessary to open the sphenoid widely, in addition to remove all fungal pieces totally. The new method described in our research could help to indicate if there are some fungal masses living in the deepest part of the sphenoid sinus. Thus, we propose a matching of two volumes: one of them is measured according to CT scan examinations, and the other one is measured intraoperatively with the use of methylene blue solution [20]. In our methodology, we present a 3D model with color using manual segmentations in CT scans. Manual segmentation provides higher precision, but requires more interaction from the operator, due to strictly hand-operated interaction. Automatic segmentation leads to a difference of up to 15% in the volume of the measured sinus as error compared to manual segmentation [34]. After automatic segmentation, the operator is required to manually examine the images, meticulously, side-image by side-image, to extract the outlines of the target structures, and make proper editing adjustments and eliminate high rates of inaccuracy. When compared, differences of CT volume and liquid measurements in the sphenoid of 0.4 cm³ were not considered due to aforementioned edema of the mucosa being present. On the contrary, if the volume difference was present, not all fungal masses were removed. This condition is common for hyperpneumatized sinuses. It is difficult for the surgeon to examine the lateral recess of the sinus where such fungal masses could still be present.

In this research the main goal was to determine a treatment strategy in patients with total opacification of the sinus. Without typical characteristics of fungal processes in the sinus or cysts in sphenoid, all patients need to be treated first without surgery; an exception to this could be patients with complicated cases or with severe headaches and no response to therapy at the first day of treatment. Our experience shows that for some patients the surgery was needed because of ineffectiveness of nasal irrigation and systemic antibiotics. In performing the retrospective and prospective analyses of medical histories and CT scans of all participants, it was found to be a combination of clinical and anatomical features that led to the treatment of choice.

The main limitation of the study is to consider only isolated bacterial sinusitis. This model is not applicable to other types of sphenoiditis.

Thus, in patients with mild or moderate clinical manifestations of ISS symptoms, and without nasal septum deviation, the probability of surgery is lower than for patients with hypoventilation of the sinus.

## 5. Conclusions

According to the statistical model presented in this study that used the reverse regression method in male patients with diffuse headache and nasal discharge, high efficiency of drug therapy (more than 78%) was shown. The presence of nasal septum deviation and adenoids in male and female patients results in the highest risk of surgical treatment (83% of the probability of the logistic model).

Patients with a history of prolonged headaches (especially diffuse headache), in addition to the presence of a one-sided decrease in vision (or progressive diplopia), CT scan examination is necessary to exclude an isolated lesion of the sphenoid sinus. In case of detection of isolated sphenoiditis, the patient requires a thorough analysis using computed tomography for the presence of prerequisites for hypoventilation of the sinus (nasal septum deviation, the presence of adenoids or concha bullosa), in addition to a survey of complaints in order to determine further treatment tactics. The combination of an isolated pathology of the sphenoid sinus and anatomical structural features of the adjacent structures, such as the nasal septum deviation and the presence of adenoids, requires immediate surgical intervention. At the same time, the combination of “headache” and “nasal discharge” symptoms in male patients is a predictor of rejection of surgical treatment in favor of conservative therapy.

## Figures and Tables

**Table 1:** Values of single-marker detection for patients with medical therapy and surgical therapy.

Anatomical Features	SEN	SPE	PPV	NPV	+LR	−LR	AUC
**Group 1 (medical therapy)**							
Gender	0.455	0.243	0.737	0.571	0.601	1.660	0.349
Nasal discharge	0.545	0.730	0.373	0.730	2.01	0.497	0.638
Nasal congestion	0.636	0.135	0.304	0.39	2.018	0.496	0.386
Headache	0.909	0.297	0.565	0.154	1.293	0.773	0.447
**Group 2 (surgical therapy)**							
Gender	0.455	0.243	0.737	0.571	0.601	1.660	0.349
Nasal discharge	0.545	0.730	0.373	0.730	2.01	0.497	0.638
Nasal septum deviation	0.091	0.568	1	0.370	0.210	4.762	0.329
Adenoids	0.136	0.757	0.404	0.750	0.560	1.786	0.447

SEN: sensitivity; SPE: specificity; PPV: positive predictive value; NPV: negative predictive value; +LR: positive likelihood ratio; −LR: negative likelihood ratio.

**Table 2:** Complaints of patients with ISS.

Patients’ Complaints	Group 1 (Medical Therapy)	Group 2 (Surgical Therapy)
Headache	19	26
Noise, hearing loss	1	0
Postnasal drip	5	15
Diplopia	0	2
Nasal congestion	4.3	4.2
Nasal discharge	14	32
Anosmia	1	1
Decreased vision	0	1
Paraorbital pain	7	6

**Table 3:** Anatomical features in the two groups of patients.

Anatomical Features	Group 1 (Medical Therapy)	Group 2 (Surgical Therapy)
Nasal septum deviation	2	16
Adenoids	3	9
Concha bullosa	0	2

**Table 4:** The microbial composition of the nose and of the sphenoid sinus were performed in patients who underwent endoscopic sphenoidotomy.

Anatomical Features	Group 1 (Medical Therapy: Systemic Decongestant, Systemic Antibacterial Therapy, Topical Glucocorticosteroids, Oral Corticosteroids and Nose Irrigation)	Group 2 (Surgical Therapy)
Staphilococcus epidermidis	11	9
Streptococcus pneumoniae	7	7
Staphylococcus aureus	2	9
Enterobacter gergoviae	2	2
Staphylococcus saprophyticus	1	1
Escherichia coli	1	0
Enterococcus faecalis	1	1
Aspergillus fumigatus	9	0
Candida No growth	2 0	0 6
